# Geographic mapping of *Enterobacteriaceae* with extended-spectrum β-lactamase (ESBL) phenotype in Pereira, Colombia

**DOI:** 10.1186/s12879-020-05267-1

**Published:** 2020-07-23

**Authors:** Deving Arias Ramos, Julián Andrés Hoyos Pulgarín, Germán Alberto Moreno Gómez, John Alexander Alzate, Juan Camilo Olaya Gómez, Isabella Cortés Bonilla, Camila Vargas Mosquera

**Affiliations:** 1 Universidad Tecnológica de Pereira, Pereira, Colombia; 2 Grupo de investigación en Medicina Interna, Universidad Tecnológica de Pereira, Pereira, Colombia; 3 Infectious diseases, Oncólogos de Occidente, Pereira, Colombia; 4 Public Health Doctor, Universidad Tecnológica de Pereira, Pereira, Colombia; 5 San Jorge University Hospital, Universidad Tecnológica de Pereira, Pereira, Colombia

**Keywords:** Geographic mapping, Geographic information systems, Antimicrobial drug resistance, Extended spectrum β-lactamase (ESBL)

## Abstract

**Background:**

Antimicrobial resistance is an ecological and multicausal problem. Infections caused by extended-spectrum β-lactamase producing *Enterobacteriaceae* (ESBL-E) can be acquired and transmitted in the community. Data on community-associated ESBL-E infections/colonizations in Colombia are scarce. Georeferencing tools can be used to study the dynamics of antimicrobial resistance at the community level.

**Methods:**

We conducted a study of geographic mapping using modern tools based on geographic information systems (GIS). Two study centers from the city of Pereira, Colombia were involved. The records of patients who had ESBL-producing *Enterobacteriaceae* were reviewed. Antimicrobial susceptibility testing and phenotypic detection of ESBL was done according to CLSI standards.

**Results:**

A population of 415 patients with community-acquired infections/colonizations and 77 hospital discharges were obtained. Geographic distribution was established and heat maps were created. Several hotspots were evidenced in some geographical areas of the south-west and north-east of the city. Many of the affected areas were near tertiary hospitals, rivers, and poultry industry areas.

**Conclusions:**

There are foci of antimicrobial resistance at the community level. This was demonstrated in the case of antimicrobial resistance caused by ESBL in a city in Colombia. Causality with tertiary hospitals in the city, some rivers and the poultry industry is proposed as an explanation of the evidenced phenomenon. Geographic mapping tools are useful for monitoring antimicrobial resistance in the community.

## Background

Antimicrobial resistance is an ecological problem that occurs worldwide and has been associated with increased resistance in both hospital-acquired and community-acquired infections [1]. Resistance in gram-negative bacteria has gained great importance in recent decades and one reason is the rapid increase of extended spectrum β-lactamase (ESBL)-producing bacteria as a growing problem worldwide [2, 3]. The Latin American region has faced a significant challenge with high levels of antimicrobial resistance among important gram-negative organisms [4]. High prevalence rates of ESBL-producing *Enterobacteriaceae* (ESBL-E) have been reported in *Escherichia coli* and *Klebsiella pneumoniae* [5]. Extended-spectrum β-lactamases (ESBLs) are β-lactamases that can hydrolyze penicillins and cephalosporins. When enteric Gram-negative bacteria acquire an ESBL gene become resistant to these classes of β-lactam agents [6]. Production of an ESBL by gram-negative bacteria is defined by reduced susceptibility to one or more of the following agents: ceftazidime, cefotaxime, ceftriaxone, cefpodoxime or aztreonam, and by potentiation of the activity of these agents in the presence of clavulanic acid [6].

Most studies on antimicrobial resistance have focused on hospital-acquired infections [7–9]. There are few descriptions of community-acquired antibiotic resistance and geographic variations in antibiotic resistance at the community level. Several studies suggest the existence of foci of antimicrobial resistance in the community [8–10]. It has been recognized that people in the community may be colonized with resistant bacteria and may behave as sources of infection [11–13]. Reports on ESBL producing *E. coli* infections occurring among patients without previous exposure to health care started to appear at the beginning of the twenty-first century [6].

The geographical variations in antimicrobial resistance have been evaluated in few investigations: six studies (4 on Gram negative and 2 on Gram positive bacteria) have been made [14–19]. The presence of specific geographic patterns of antimicrobial resistance appears to be a common finding in these studies. As we learned that ESBL-producing *Enterobacteriaceae* can be acquired and transmitted in the community, we wondered if there may be geographic patterns that show ESBL-E community transmission areas and if we can find ecological factors that explain them. Two studies have demonstrated the presence of ESBL-producing *Enterobacteriaceae* clusters at the community level using geographic mapping tools [14, 15]. The ESBL-E dissemination is of paramount importance because the associated antimicrobial use has a potential to further select for even more resistant pathogens such as carbapenem-resistant *Enterobacteriaceae* [2, 20]. We hypothesized that there are specific geographic areas of antimicrobial resistance in the community, and due to the high prevalence of ESBL-producing *Enterobacteriaceae* reported in Latin America, we decided to conduct a geographic mapping study. Modern georeferencing tools offer the possibility to geographically locate and analyze patients with resistant bacteria [21].

This study was conducted in the city of Pereira, Colombia. Pereira is a Colombian municipality, capital of the department of Risaralda. The city is located in the central-western part of the country in the Central Cordillera of the Colombian Andes. Pereira has a population density of 841.25 inhab / km^2^ and is the most populous city in the agro-ecological region of coffee production (also known as the Coffee Triangle) with an approximate population of 409.670 inhabitants [22] and it is divided into communes. The communes are delimited geographical areas in the urban area of the city.

## Methods

We conducted a retrospective study at the “San Jorge” University Hospital (SJUH) and the “López Correa” Clinical Laboratory (LCCL) to investigate cases of ESBL-E infections and colonizations from January 2012 through December 2017. Cases were identified from the microbiology laboratory database of both centers, followed by review of electronic patient medical records stored in SJUH database. Demographic information were extracted using standardized data collection sheets. The “San Jorge” University Hospital is a major public hospital in Pereira. It is equipped with over 300-beds. The López Correa Clinical Laboratory (LCCL) is a major laboratory that processes mainly outpatient consultation samples and has several offices in the city, both centers are located in the city of Pereira, Colombia. The study was approved by the Ethics Committee of the *Universidad Tecnológica de Pereira*.

To establish the geographical distribution, the QGIS 3.4.11 software was used, a geo-referencing software based on geographic information systems (GIS) [23]. QGIS is a free and open source geographic information system. Digitized maps of the city (Pereira) were also used.

### Definitions

It was determined if the patients had an infection or colonization by an ESBL-E and also, if the infection or colonization was community-acquired or hospital-acquired. For this purpose, several criteria were used [24–27]:

- Colonization was considered when the sample came from non-sterile tissue and the patient had no associated symptoms of clinical infection, eg, asymptomatic bacteriuria, colonization of rectal mucosa.
- An infection was considered when the sample came from a sterile or non-sterile tissue, and the patient had associated symptoms of clinical infection, eg, urinary tract infection, peritonitis, intra-abdominal abscess.
- An infection / colonization was considered community-acquired if the sample was taken from outpatient consultation or if the patient was admitted to a hospital and had an ESBL-E in a clinical sample that was taken within 48 h of hospital-admission.
- An infection / colonization was considered hospital-acquired if the clinical sample was taken from the patient after 48 h of hospital-admission.

Based on these statements, the following patient groups were established:

- Community acquired infection/colonization: an ESBL-E identified in a clinical sample that was taken by outpatient consultation or within 48 h of being admitted to the SJUH with clinical signs of infection (community acquired infection) or no clinical signs of infection (community acquired colonization).
- Patients with hospital acquired infection / colonization, discharged alive: all inpatients who had an infection / colonization that was considered hospital-acquired and were discharged alive.

### Data collection and microbiological studies

The microbiology laboratory of each center provided patient records. To obtain patient addresses at the townland level, each patient record was reviewed. ESBL detection was done by automated method. Antimicrobial susceptibility testing and phenotypic confirmation of ESBL were performed according Clinical Laboratory Standards Institute (CLSI) recommendations. In the SJUH Vitek 2 system (bioMérieux SA) was used. In LCCL the BD Phoenix system (Becton and Dickinson) was used until 2015, from 2015 onwards the Vitek 2 system was used. Minimum inhibitory concentration (MIC) profile was used as a screening test for ESBL. Screen positive isolates (MIC of cefotaxime or ceftazidime of ≥1 mg/L, an ESBL or inducible cephalosporinase warning by the automated system) were subjected to confirmation tests using double disc synergy method for ESBL. All isolates showed sensitivity to carbapenems according to the cutoff points for each year by the CLSI.

### Geocoding, mapping

To perform the geographic mapping we extracted the home address from the patient records. QGIS 3.4.11 was used to create, edit, visualize and analyze geospatial information. This software requires a Shapefile-type data map. To create the shape-file, we obtained the coordinates of the home address of each patient through a manual search on Google® Maps. To ensure the compatibility of Colombian coordinates with spatial positioning techniques we had to use MAGNA SIRGAS PRO-3.0, a software based on the geocentric reference system for the Americas [28]. With the information of the coordinates (latitude and longitude), the Shapefile-type data maps were created using the GIS software [23].

Heatmaps were created using the QGIS *Heatmap kernel density estimation* (KDE) tool to obtain visual information of the areas of greatest concentration of events. KDE helps identify the presence of clusters and irregularities [29]. This tool requires a radius to determine the circular neighborhood around each point, where that point will have influence, otherwise speaking, KDE determines how the influence of a point extends in the given radius [14, 16, 19]. We arbitrarily assume that each home address of a given patient could have a radius of influence of 250 m.

The city’s communes and rivers are shown on each map. This information was obtained from the website of the geographical portal of Pereira [30]. A layer of “*OpenStreetMap*” is also displayed in the background. Based on the address of each tertiary hospital in the city, a geographic mapping was made of each one, a total of three are shown on the maps in Fig. 2.

### Statistical analysis

Baseline characteristics were compared using Chi-square or Fisher’s exact tests where applicable for categorical variables and independent t-test for continuous variables. Variables with *p*-value of < 0.05 were considered significant. IBM SPSS Statistics software version 20 was used for all statistical analyses.

## Results

We obtained a database of 11.230 and 11.340 cultures with gram-negative bacilli from the “San Jorge” University Hospital (SJUH, group 1 mainly inpatient) and the “López Correa” Clinical Laboratory (LCCL, group 2, mainly outpatients), respectively. The ESBL-E prevalence was 8.5% (*n* = 965) and 11.1% (*n* = 1262) respectively.

**Fig. 1:**
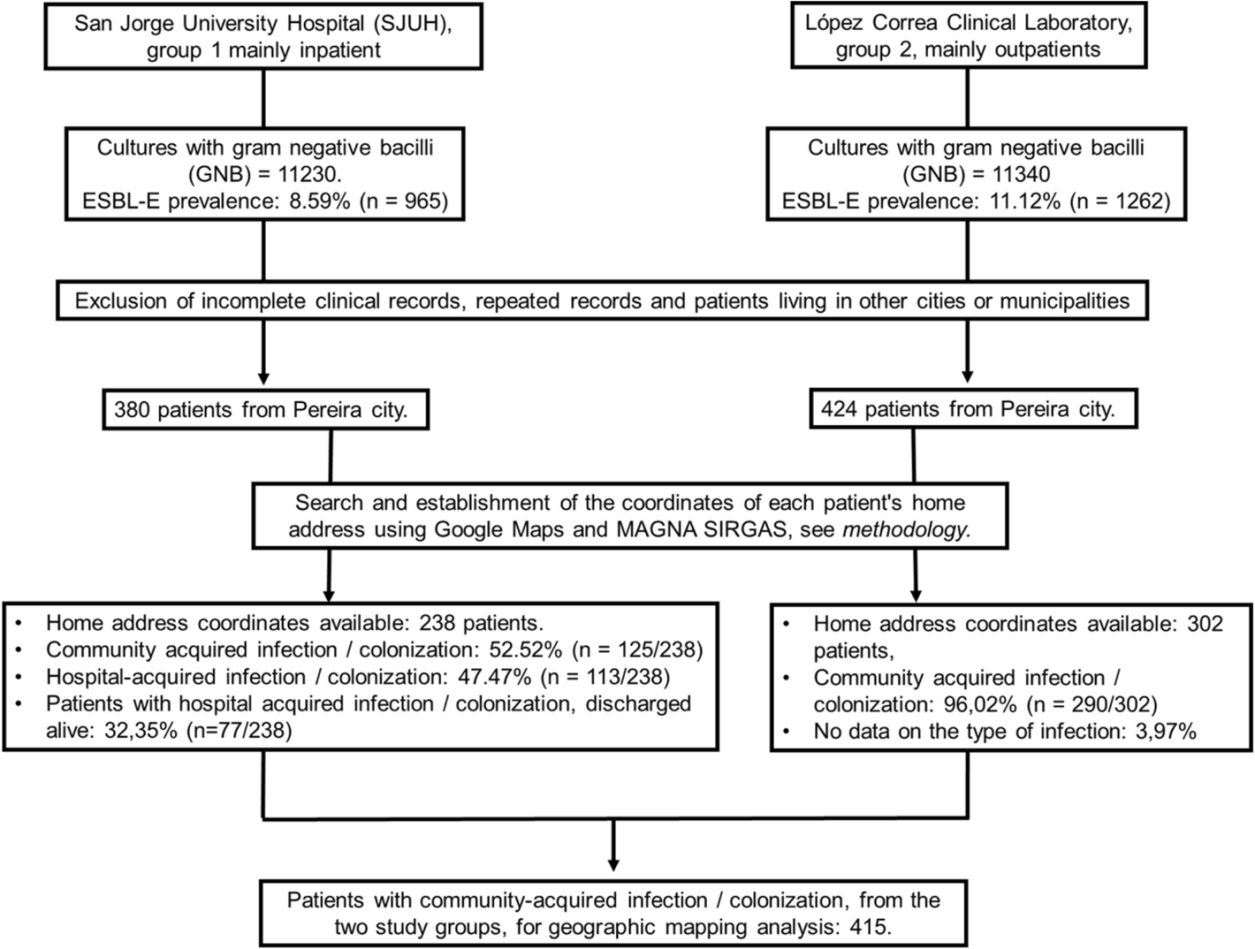
Flow chart for patient selection from study centers

We reviewed all records and excluded incomplete clinical records, repeated data, and patients living in other cities or municipalities. Figure 1 shows the flow chart of the study patients. Eight hundred four patients lived in the city of Pereira. Following the proposed methodology, we were able to obtain the home address coordinates of 238 and 302 patients who were seen at SJUH and LCCL, respectively. Upon review of the records, it was determined that 52.5% (*n* = 125/238) and 96.0% (*n* = 290/302) of SJUH and LCCL patients had a community-acquired infection/colonization, respectively. Therefore, a total of 415 patients were available for analysis. The mean age was 65.72 years, only 3.8% (16 patients) were under 18 years old, most patients were over 50 years (83.6%) and 65.3% were women. Table 1 shows the clinical and microbiological characteristics of the patients and the *P* value for differences between the two study groups. Both groups are heterogeneous. The most common source of sampling was the urinary tract, representing 80 and 100% of the samples in SJUH and LCCL patients, respectively. Group 1 patients had urinary tract infection in 40% (*n* = 50/125) and another 40% had asymptomatic bacteriuria.

**Table 1:** Clinical and microbiological characteristics of patients with community-acquired infection / colonization by ESBL-E

	Community-acquired infection / colonization *n* = 415	SJUH. Group 1 mainly inpatient, *n* = 125	LCCL. Group 2, mainly outpatients *n* = 290	*P*	CI95%
Female n(%)	271 (65,3)	52 (41,6)	219 (75,5)	0,000	0,24 to 0,43
Male n(%)	144 (34,7)	73 (58,4)	71 (24,4)	0,000	-0,43 to - 0,24
Mean age	65,72	67,03	65,17	0,403	−6,25 to 2,51
≥50 years old n(%)	347 (83,6)	107 (85,6)	240 (82,7)	0,145	−0,10 to 0,05
Sampling site					
Outpatient consultation n(%)	292 (70,4)	2 (1,6)	290 (100)	0,000	0,96 to 0,99
Inpatient n(%)	123 (29,6)	123 (98,4)	0	0,000	−0,99 to - 0,96
*E.coli* n(%)	304 (73,3)	74 (59,2)	230 (79,3)	0,000	0,11 to 0,29
*K.pneumoniae* n(%)	102 (24,6)	50 (40)	52 (17,9)	0,000	−0,30 to - 0,13
Urine sample n(%)	390 (94)	100 (80)	290 (100)	0,000	0,15 to 0,24
Non-urinary sample n(%)	25 (6)	25 (20)	0	0,000	−0,24 to - 0,15

*SJUH* San Jorge University Hospital, *LCCL* López Correa Clinical Laboratory. The value of *P* is shown for the differences between group 1 and group 2

On the other hand, 113 patients in group 1 had hospital acquired infection/colonization. Patients with hospital acquired infection/colonization discharged alive were 68.1% (*n* = 77/113). This group of patients had a variety of infections, to mention, urinary source (37 patients), bacteremia (14 patients), abdominal infection (7 patients) and soft tissue infection (7 patients). *E. coli* was identified in 57.1% (*n* = 44/77) and *K. pneumoniae* was identified in 41.5% (*n* = 32/77).

Community acquired ESBL-E.

**Fig. 2:**
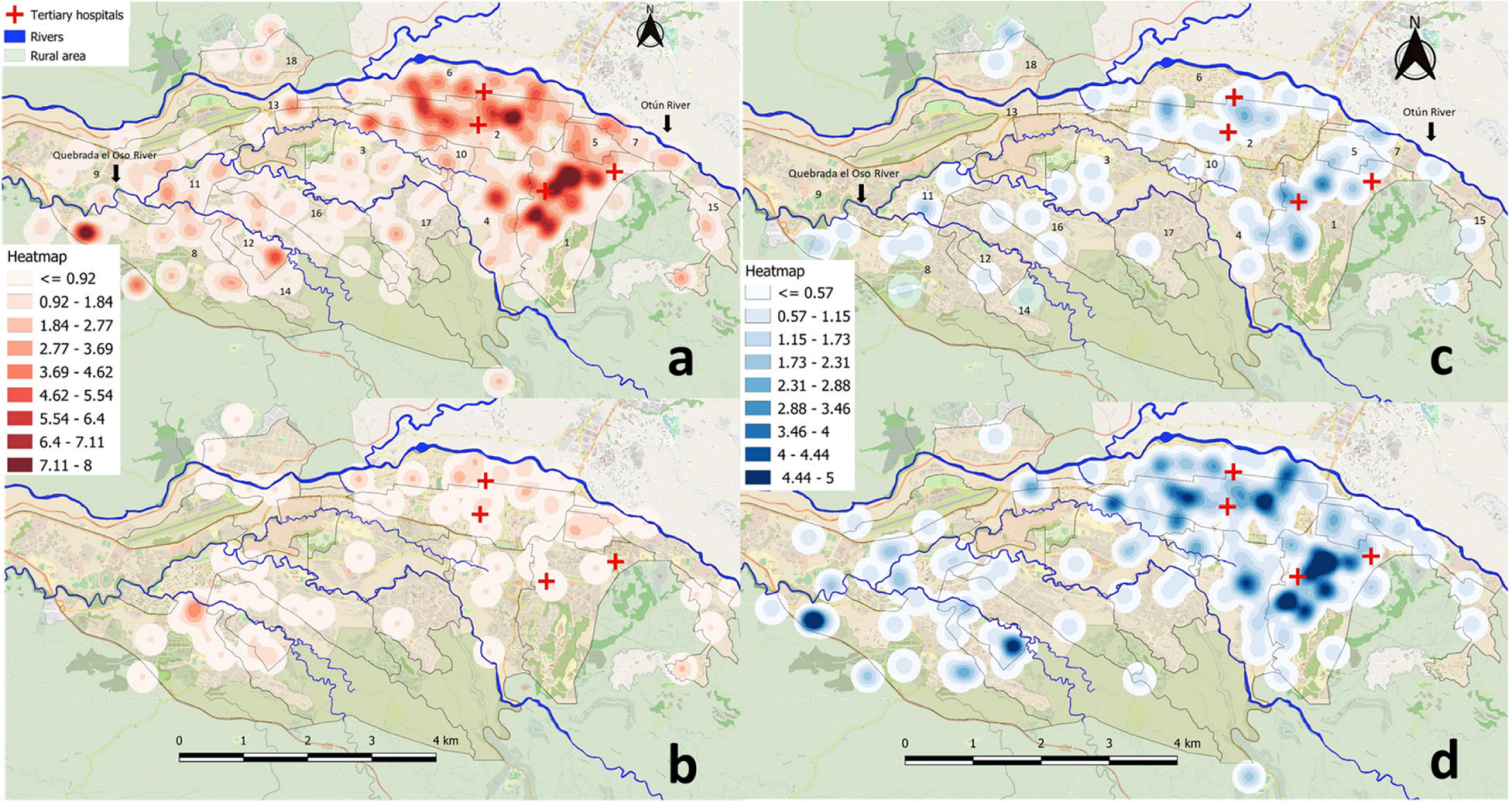
Heat maps of the city of Pereira, Colombia. **a** Community-acquired infections/colonizations from January 2012 through December 2017, population analyzed 415 patients. **b** Patients with hospital-acquired infections/colonizations discharged alive from the tertiary hospital, from January 2012 through December 2017, population analyzed 77 patients. **c** Community-acquired infections/colonizations in 2012–2013, population analyzed 81 patients. **d** Community-acquired infections/colonizations in 2016 to 2017, population analyzed 233 patients. The city’s tertiary hospitals, communes, and rivers are shown on each map. Each commune is delimited by black lines and are numbered from 1 to 18. The maps depicted in Fig. 2 is the product of our own work. The QGIS software was used for this purpose. QGIS is a Free and Open Source Geographic Information System. A layer of “OpenStreetMap” is also displayed in the background, this is a Free and Open Source Geographic Information to use under an open license. The maps of the communes and rivers were obtained from the website of the “geographical portal of Pereira” where access to geographic information is provided as a Free and Open Source Geographic Information

The map in Fig. 2a shows the geographical location of the ESBL-E originated at the community level. This map was created with a heterogeneous population of 415 patients and show an overview of the city of Pereira where the urban area is highlighted. The communes are delimited by black lines. The rivers are represented by blue lines and the main tertiary hospitals are represented by red crosses. The *Otún* River is the main nearby river and crosses the north of the city. A higher concentration of patients is observed in the urban area, forming some clusters. Several hotspots in the south-west and northeast areas of the city are shown (Fig. 2a). The main communes are numbered. Their local names appear in Table 2. Hotspots are seen in the east of the city in communes 1 to 7, 10 and 15, and also in the north-west in communes 13 and 18. Some of these communes are close to the *Otún* River (Fig. 2a). The flow of the river goes from east to west and it is important to mention that in the east of the city, in the rural area, there are different settlements of the poultry industry near the river bank (not shown in the Fig. 2). Hotspots are also seen in the western region of the city in the communes number 8, 9 and 11 to 14. Some of these communes are near several rivers, the most prominent in this area named “Quebrada el Oso” (Fig. 2a).

**Table 2:** Prevalence of community-acquired ESBL-E per commune

Commune, number.	Commune, name.	Inhabitants per commune	Community-acquired ESBL-E *n* = 392	Prevalence per 10,000 inhab.
1	Universidad	16919	84	49,6
2	Centro	32663	77	23,5
3	El Jardín	10652	18	16,8
4	Boston	23925	31	12,9
5	Villavicencio	12991	16	12,3
6	Río Otún	38699	42	10,8
7	Oriente	16437	15	9,1
8	San Joaquín	29984	27	9,0
9	Olímpica	14146	12	8,4
10	San Nicolás	8635	7	8,1
11	Cuba	18604	11	5,9
12	Perla Del Otún	13816	8	5,7
13	Ferrocarril	10456	6	5,7
14	El Oso	23669	12	5,0
15	Villa Santana	16569	7	4,2
16	Consotá	21775	8	3,6
17	El Poblado	17742	6	3,3
18	Del Café	25203	5	1,9
	Total	352885	392^a^	

^a^392 were included to estimate the prevalence per / commune. The remaining 23 patients lived in the rural area

Among all patients, 94.4% (*n* = 392/415) lived in the urban area of the city distributed in 18 communes of the city. The remaining 23 patients lived in rural area. Most community-acquired ESBL-E patients were from some highly populated communes, to mention, commune 1, 2, 4, 6, 8 and 11. We obtained data on the inhabitants by commune from a query to the National Administrative Department of Statistics of Colombia [31], then, the prevalence of ESBL-E of each commune was established. Table 2 shows the prevalence of community-acquired ESBL-E for each commune. The communes with the highest prevalence of ESBL-E were also those with the most prominent hotspots in Fig. 2a.

### Patients with hospital acquired infection/colonization

Seventy-seven patients were discharged alive from the San Jorge University Hospital, where they had a hospital-acquired ESBL-E infection / colonization. A map was created to see the geographical areas where these people live. Figure 2b shows in a striking way that the geographical areas of hospital discharges coincide with some geographical areas where there were community-acquired ESBL-E. This could represent the mobilization of antimicrobial resistance from hospitals to the community. The presence of tertiary hospitals in communes 1, 3 and 6 should also be highlighted, as they could be sources of antimicrobial resistance in the community.

### Changes through time

We compared the number of cases in two time periods, one in the years 2012–2013 (*n* = 81/415, Fig. 2c) and the other one in the years 2016–2017 (*n* = 233/415, Fig. 2d). We found a statistically significant difference in the number of cases between the time periods (19.5% vs. 56.1% respectively, *p* = 0.000, CI95% 0.21–0.31). This difference is also observed in the maps in Fig. 2c and d corresponding to the years 2012–2013 and 2016–2017 respectively. The intensity of the hot-spots is higher in the 2016–2017 period.

## Discussion

The present work shows hotspots that indicate the community transmission of ESBL-E in the urban area of a city in Colombia. Therefore, it is suggested that there are ecological factors that explain the presence of foci of antimicrobial resistance at the community level. The present study shows how GIS can be applied for local-scale zoning of high-risk areas of antimicrobial resistance. But, even so, it is not enough, at least in our work, to establish causality and determine the ecological factors causing these clusters of antimicrobial resistance. ESBL-E community transmission is likely multifactorial [7, 9, 11, 12, 32, 33], but potential contributing sources include animals (both food and companion), the environment and direct transmission within households [6]. George et al., in a study with 363 stool samples from healthy subjects in the district of Vellore, South India, identified clusters of patients and healthy individuals with ESBL-E near sewage drains and public toilets, suggesting the acquisition of ESBL-producing *E. coli* from environmental sources [15]. Norman et al. identified 1399 subjects with ESBL-E infection living in the metropolitan area of Leeds and Bradford from samples that were taken at the University Hospital of Leeds and the University Hospital of Bradford and also isolates originated at the community level. The authors found clusters of ESBL-E in some areas within Bradford, without a clear explanation of the phenomenon [14].

The community origin of the ESBL-E has been demonstrated elsewhere [15]. CTX-M enzymes are worrisome as they have the potential to spread outside the hospital environment. *E. coli* is the main producer of β-lactamases (*Bla*) of the CTX-M type behaving like a community pathogen. On the other hand, *Klebsiella* spp. is more frequently associated with hospital-acquired infections, produces *Bla* of the TEM and SHV type and is more frequently found causing respiratory infections, intra-abdominal infections and bacteremia [34].. *E. coli* causing urinary tract infections or asymptomatic bacteriuria was, as expected, the most frequent bacteria in the present work. However, the proportion of patients with *K. pneumoniae* in group 1 was higher than group 2, (40% vs. 17% *p* = 0.000, 95% CI-0.309 to − 0.132) despite having both community-acquired infections/colonizations.

There are several ecological factors that explain the community transmission of ESBL-E. As mentioned before, George et al [26] found that the contact with open sewage drains and public toilets could explain ESBL-E foci at the community level. As geographic mapping can assist in hypothesis formulation, we think of two hypothesis: (i) the interaction between tertiary hospitals and the community and; (ii) nearby rivers can be a source of antimicrobial resistance in the community because, it has been mentioned that the natural aquatic environment can be a contributor to the development and circulation of antibiotic resistance genes [35]. *Otun* River is highlighted as it is used for recreational purposes and settlements of the poultry industry are near the river bank. Other well-documented factors associated with community-acquired antimicrobial resistance, such as contact with nursing homes [19] and antibiotic consumption at the community level [18] were not evaluated in our work. Additional research is required to determine the ecological factors and establish mechanisms to control them.

The changes in the density of cases through time, as seen in the heatmaps (Fig. 2c and d), demonstrate that antimicrobial resistance is a continuously evolving process and that the monitoring of geographic patterns is important to detect outbreaks. This evidence supports the fact that there is a steady and continued increase of ESBL-producing *Enterobacteriaceae* infections and that this phenomenon has gone unnoticed in our region. These data suggest that geographic mapping tools can be used in monitoring the phenomena of antimicrobial resistance in the community.

GIS can be used to identify a subset of patients at high risk for community-acquired ESBL-E when certain geographic areas of the city are involved. However, we don’t know to what extent living in a geographic area with a high prevalence of ESBL-E could be considered a risk factor for developing an infection caused by this type of bacteria or; to what extent this information should compel us to use an empirical treatment against ESBL-E when a patient living in these geographical areas seeks care for a community-acquired infection, for example, a urinary tract infection. An approach like this has been suggested by other authors [17]. It is well known that the associated antimicrobial use of carbapenems has a potential to further select for even more resistant pathogens such as carbapenem-resistant *Enterobacteriaceae*. Therefore, this information must be adequately weighted. Appropriate empirical therapy has been an issue worldwide, especially with the progressive increase in antimicrobial resistance, where careful and wise choice of antibiotics is necessary and should be supported by antimicrobial stewardship.

The spread and increase of ESBL-E is a fact, also the transmission in the community has been demonstrated. The health care facilities, the food industry, international / domestic travel and the environment are involved in the spread of ESBL-E. Antimicrobial treatment options are limited and have failed to keep pace with increased antimicrobial resistance. So, just as we do not have better treatment options, we must reinforce other control strategies in the battle against antimicrobial resistance. Antibiotic Stewardship Programs (ASPs) have been useful in optimizing the prescription of antibiotics in hospitals, and if it works for hospitals, we could transfer it to the community to control antibiotic prescriptions, pharmacy sales, the consumption of antibiotics in the food industry, surveillance of resistance genes and resistant bacteria in water sources and monitor nursing homes. The tools we use for this purpose are justified, and thus, GIS are a novel detection and monitoring tool that can help us to identify foci of transmission in the community and to contain as much as possible the spread of antimicrobial resistance. A combined approach that involves not only control measures in healthcare facilities but also in the community and public health is required to control spread of ESBL-E.

This study has a number of limitations. First, the prevalence of ESBL-E was calculated based on the data of only two main health centers of the city. No all the events of ESBL-E infection / colonization could be included and thus we were unable to measure the prevalence with a high level of confidence. Second, the study design did not allow us to determine causation or directionality of association between ESBL-E and commune or district–level characteristics. Third, we depended on data recorded in two health care facilities. All patients with ESBL-E may not visit the health care facilities and thus the data presented may be an underreporting of the real data in the city. On the other hand, there are no larger ESBL-E databases in our region. Fourth, since the study was based on two disparate institutions data, the study groups are heterogeneous and some important demographic and clinical variables were not available in the reports and therefore were not included. The hospital expectedly has both in- and out- patients whereas the reference laboratory center caters only to outpatients. We had limitations to establish the clinical characteristics of the outpatients of the reference laboratory center (group 2): 100% of the clinical samples in group 2 were from urine, but we were unable to determine whether they had a urinary tract infection or asymptomatic bacteriuria. Fifth, only population that had specific infection or colonization by ESBL-E was included. We did not make a comparison with another gram-negative resistance mechanism, nor with sensitive gram-negative bacteria. Sixth, the presence of ESBL was established by conventional laboratory methods and no genotypic confirmation was performed.

## Conclusions

The existence of antimicrobial resistance clusters by ESBL-E at the community level in a city in Colombia was demonstrated. We infer that the presence of tertiary hospitals and recreational activities in rivers close to the city and close to the poultry industry could be ecological factors that explain this mechanism of bacterial resistance in the community. However, the ecological factors associated with antimicrobial resistance could not be sufficiently evaluated and further studies are required.

GIS are innovative and important component in studies of public health and epidemiology. Geographical mapping tools are useful for monitoring antimicrobial resistance in the community. An increase in the density of hot spots was demonstrated over time, but the factors associated with these findings are unknown.

Exploring the relationship between ESBL-E cases and environmental factors should be further investigated to minimize risks of disease transmission at the community level.

## Data Availability

The datasets used and/or analyzed during the current study are available from the corresponding author on reasonable request.

## Abbreviations

SJUH: San Jorge University Hospital
LCCL: López Correa Clinical Laboratory
ESBL: Extended spectrum β-lactamase
ESBL-E: Extended spectrum β-lactamase producing *Enterobacteriaceae*
Bla: β-Lactamase
UTI: Urinary tract infection
CLSI: Clinical & Laboratory Standards Institute
